# Comparison of seven single cell whole genome amplification commercial kits using targeted sequencing

**DOI:** 10.1038/s41598-021-96045-9

**Published:** 2021-08-25

**Authors:** Tamir Biezuner, Ofir Raz, Shiran Amir, Lilach Milo, Rivka Adar, Yael Fried, Elena Ainbinder, Ehud Shapiro

**Affiliations:** 1 Department of Computer Science and Applied Mathematics, Weizmann Institute of Science, 234 Herzl Street, 7610001 Rehovot, Israel; 2 Stem Cell Core and Advanced Cell Technologies, Life Sciences Core Facilities, Weizmann Institute of Science, 761001 Rehovot, Israel

**Keywords:** PCR-based techniques, Next-generation sequencing, Targeted resequencing, Computational biology and bioinformatics

## Abstract

Advances in whole genome amplification (WGA) techniques enable understanding of the genomic sequence at a single cell level. Demand for single cell dedicated WGA kits (scWGA) has led to the development of several commercial kit. To this point, no robust comparison of all available kits was performed. Here, we benchmark an economical assay, comparing all commercially available scWGA kits. Our comparison is based on targeted sequencing of thousands of genomic loci, including highly mutable regions, from a large cohort of human single cells. Using this approach we have demonstrated the superiority of Ampli1 in genome coverage and of RepliG in reduced error rate. In summary, we show that no single kit is optimal across all categories, highlighting the need for a dedicated kit selection in accordance with experimental requirements.

## Introduction

The increase in throughput and precision of next generation sequencing (NGS) in recent years had a dramatic effect on biological research. Cell to cell variability within the same organism became a highly investigated research field, underlying the need for new and improved molecular biology analysis tools. Such variability may be in multi cell properties (e.g. gene expression, genomics, epigenomics and proteomics)^1^. SC genome variability is a fascinating example for the need for accurate measurements, as sequence variations occur during development in health (e.g. random somatic mutations, genomic recombination during B and T cell maturation) and disease (e.g. driver mutations and copy number alterations in cancer).

Since single-molecule-sequencing is still in its early stages, a variety of whole genome amplification (WGA) protocols, which amplify the entire genome make the current state-of-the-art in SC genome analysis. A genome contains a single copy of each nucleotide and hence, any bias, biochemical or computational, that may lead to modification or loss of information will have a dramatic effect on the conclusion of an experiment^2^. Biochemical biases may occur mainly due to damaged cells or by amplification bias. Examples for such bias are in vitro mutation, loss of genomic regions (allelic drop out-ADO) and non-uniform amplification that may disrupt copy number variation (CNV) analysis or lead to ADO in cases of shallow NGS coverage. The reproducibility of the protocol is sometimes more important than the examples above, for example when SC sequences are compared^3^).

WGA protocols differ by various parameters, namely by the polymerase type, and the molecular biology principles standing behind the amplification (as reviewed here^2,4^). WGA protocols originally emerged to enable the analysis of low starting DNA material, and in recent years, SC (containing ~ 6 pg of DNA) dedicated WGA kits (scWGA) were developed and commercialized.

Although several scWGA kit comparisons were published^5–12^, none has yet to compare all of the available kits in a single comparison, and at most, selected kits that represent the same category were selected for comparison. The goal of this study is to compare all commercially available scWGA kits (known to the authors to the date of the experiment design, Table 1), by using a previously established targeted sequencing approach^3^. In summary, we processed 12–23 normal SCs using seven different kits (125 SCs in total), and analyzed them using a panel of 3401 amplicons, composed mainly of short tandem repeats (STR) regions. The following aspects were analyzed: genome coverage, reproducibility of amplification between SCs (intersecting successfully amplified loci) and, due to the instable nature of STR in vitro amplicon, the error-rate of each scWGA kit.

**Table 1:** Summary of participating scWGA kits.

Kit short in manuscript	Kit name	Cat. number	WGA technique	Company	Cleanup?	Final reaction volume (µl)	Elution volume after cleanup(µl)	Number of analyzed cells	Cost per reaction ($)
Ampli1	Ampli1 WGA Kit	WG 001 050 R02	Linker adapter PCR (LA-PCR)	Silicon biosystems	–	52	–	22	28
MALBAC	MALBAC Single Cell WGA Kit	YK001B	Multiple Annealing and Looping Based Amplification Cycles (MALBAC)	Yikon genomics	MinElute (Qiagen)	65	35	18	35
GenomePlex	GenomePlex Single Cell Whole Genome Amplification Kit (WGA4)	WGA4	Degenerate oligonucleotide-primed PCR (DOP-PCR)	Sigma	GenElute (Sigma)	75	50	13	24
PicoPlex	PicoPLEX WGA Kit	E2620S	Displacement DOP-PCR (D-DOP-PCR)	New England Biolabs (manufactured by Rubicon)	MinElute (Qiagen)	75	35	19	36
GPHI-SC	illustra Single Cell GenomiPhi DNA Amplification Kit	29-1081-07	Multiple displacement amplification (MDA)	GE Healthcare	Ethanol precipitation (in accordance with protocol)	30	35	12	30
RepliG-SC	REPLI-g Single Cell Kit	150345	MDA	Qiagen	–	50	–	23	24
TruePrime	TruePrime Single Cell WGA kit	350025	MDA	SYGNIS	QIAquick (Qiagen)	45	35	18	15

## Results

### Generation of a large cohort of single cells data for scWGA kits comparison

In order to create a comprehensive analysis of scWGA kits we aimed to pick and analyze a uniform population of cells (originated from the same clone) using all commercially available scWGA kits (Table 1).

We generated a clone from a single human ES cell (H1) that is considered normal without known chromosomal aberrations. Following clonal expansion cells were dissociated to enable SC picking using an automated cell picker CellCelector (ALS). Cells were picked to scWGA dedicated 96 well PCR plate, pre-filled with a kit dedicated deposition buffer. Cells were then processed by different scWGA kits (see “Methods”). Following scWGA, DNA samples were randomized and processed by our previously published amplicon based targeted sequencing protocol, using AccessArray microfluidics chips (herein: AA, Fluidigm)^3^. Overall, the amplicon panel is comprised of 3401 amplicons (Supplementary Dataset 1), 95% of the amplicons in the panel comprise of 4282 STR loci (Supplementary Dataset 2). Following shallow sequencing we analyzed (a) coverage per amplicon per sample, and (b) sample success rate (mapped reads/total reads) (Supplementary Dataset 3).

We first opted to validate the robustness and accuracy of the analysis platform by: (1) validation of PCR negative and positive controls (inserted directly to the AA chips) by analysis of success rate and % mapped amplicons (Supplementary Fig. 1a,b, respectively); (2) the replication of the same analyses as in (1) for 40 DNA duplicates distributed randomly across AA chips (Supplementary Fig. 1c,d, respectively). We then normalized the sample volumes to give equal read counts in deep sequencing run (see “Methods”). A single replicate was randomly selected from each cell sample duplicates (noted in Supplementary Dataset 3).

### scWGA genome coverage analysis

**Figure 1:**
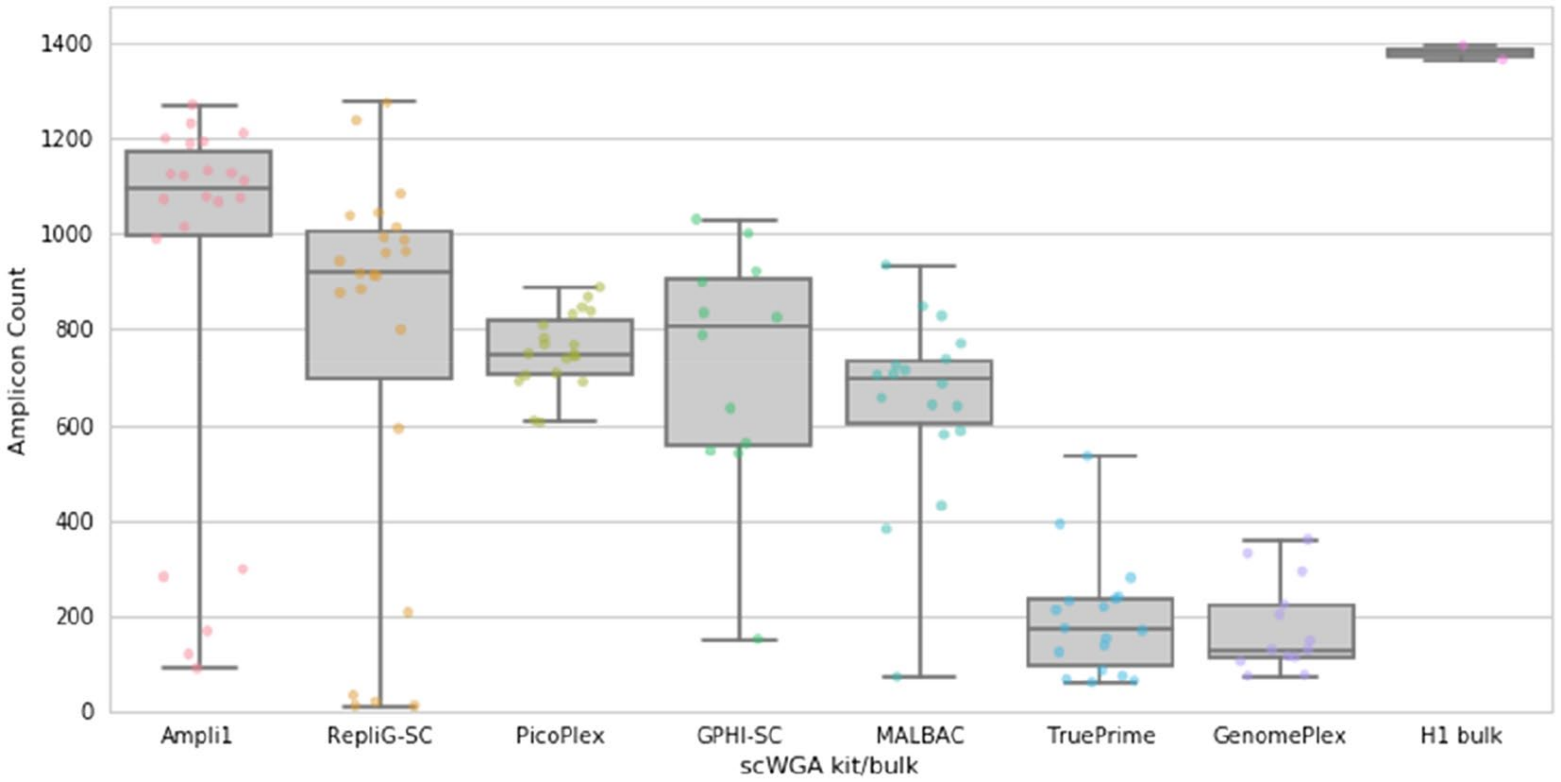
Amplicon coverage per single cell kit of only X chromosome “TTAA” free amplicons. Mapped amplicons were counted per each single cell. Data represents only MseI restriction site free amplicons (“TTAA”) in the X chromosome (see Supplementary Dataset S3), to follow the internal bias of the Ampli1 against these amplicons (see Supplementary Fig. 2). Each dot represents a single cell, except for the right column, where each dot represents a cell bulk duplicate, originated from the same cell line (H1). Each column is the collection of all single cells per scWGA kit (except for the H1 bulk column). The theoretical maximum is 1912 amplicons that follow the X chromosome, TTAA free criteria. However, 327 amplicons failed to amplify in all samples, making the practical maximum number of working amplicons for this analysis 1585.

One of the key measurements in SC genome research is the genomic coverage. Poor genome coverage is namely a result of biochemical biases and faulty amplification. Using our amplicon panel, we sought to validate the genome coverage by counting the number of successful amplicons (> 0 reads). Improper amplification may lead to misinterpretation of the data for genome coverage analysis. For example, if a single allele is not amplified, it might be undetected, as the other allele may “compensate” for the loss. To tackle this we first filtered and counted only amplicons on the X chromosome (2495 amplicons, 73% of the full panel). In addition, Ampli1 kit is based upon genomic digestion of the MseI restriction enzyme (sequence recognition site “TTAA”) and hence, amplicons that contain MseI (583 in the X chromosome, comprise 17% of the full panel) will not be amplified by this kit (Supplementary Fig. 2). Hence, a fair comparison between kits would exclude all amplicons that include MseI recognition regions (Supplementary Dataset 1). The theoretical number of amplicons that correspond to the above criteria (X chromosome, TTAA free amplicons) is 1912 (56% of the panel). Notably, out of these amplicons, 327 failed to amplify in all samples, making the *practical maximum number* of working amplicons for this analysis 1585 (47% of the full panel). Plotting all SCs per kit in a single graph (Fig. 1) shows that Ampli1, and later RepliG-SC are the best at the genome coverage aspect, yielding medians of 1095.5 and 918 amplicons per SC, respectively. GPHI-SC, PicoPlex, and MALBAC are next in this category with medians of 807.5, 750 and 696.5 amplified loci per SC, respectively. Notably, PicoPlex is the most reliable kit, with the tightest inter quartile region (IQR) of all kits, and no failed cell. Specific experimental calibrations may assist in reducing the failed cells (improvements of picking, modifying the protocols etc.). GenomePlex and TruePrime generated significantly less mapped reads (Supplementary Dataset 3) and as a result, lower number of mapped amplicons. Their positive control results are similar to the SC data, leading to the conclusion that the poor results are not due to cell picking procedure but a failure in kit performance.

### scWGA reproducibility analysis

**Figure 2:**
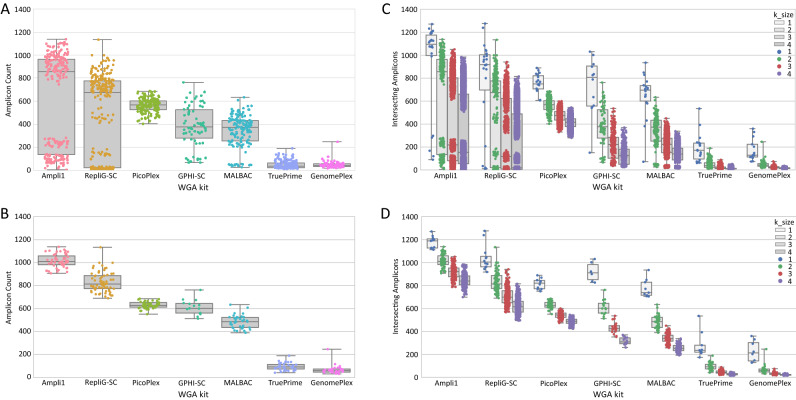
scWGA reproducibility analysis. (**A**) Each pair of cells (k = 2) per scWGA kit were analyzed for the number of mapped amplicons that were mapped in both cells. Each dot represents a pair of cells, y-axis is the number of amplicons which were mapped by > 0 reads that worked for both cells (intersecting amplicons). (**B**) Same analysis as in (**A**) but for the upper median of the most successful cells as reflected by their number of amplifying amplicons (Fig. 1). (**C**, **D**) Same analysis as in (**A**) and (**B**), respectively for cell groups comprised of k = 1 to k = 4 cells (k = 1 is the same as presented in Fig. 1, k = 2 is the same as presented in (**A**) and (**B**). Repeated presentation is for visualization of results in the context of all group sizes).

In some cases, the reproducibility of the scWGA kit over several SC samples is more important than other important parameters (such as a genome coverage). In order to analyze reproducibility, we have generated a dataset of all possible groups of cell pairs (not duplicates) per kit and counted the number of intersecting loci for all calculated groups, with the restriction of X chromosome, MseI free regions, as explained above. Results (Fig. 2a) demonstrate that Ampli1 is the most reproducible kit, having more intersecting loci than its follower, RepliG-SC. Notably, we can see clusters of cell groups that partially or completely failed. To get a better simulation of a real experiment, where successfully amplified cells are selected for analysis, we have selected the cells from the upper median amplicon coverage per kit, as presented in Fig. 1 and performed similar reproducibility analysis (Fig. 2b). As expected, results demonstrate a tighter range of intersecting amplicons and demonstrate Ampi1 superiority. A repeat of the same type of analysis for groups sizes of k = 3 and 4 cells shows similar results (Fig. 2c,d), with an expected drop of intersecting amplicons as cell group size increases. Interestingly, analysis shows a mild decrease in the number of intersecting loci as the group size was increased for all kits. This provides a strong evidence that WGA protocols are systematically biased for the loci they amplify from the genome. PicoPlex, although not the best in the aspect of amplicon coverage, when compared to other kits, demonstrates high reproducibility for all of its cells (Fig. 2c) supporting the biased amplification assumption.

### scWGA error rate analysis

**Figure 3:**
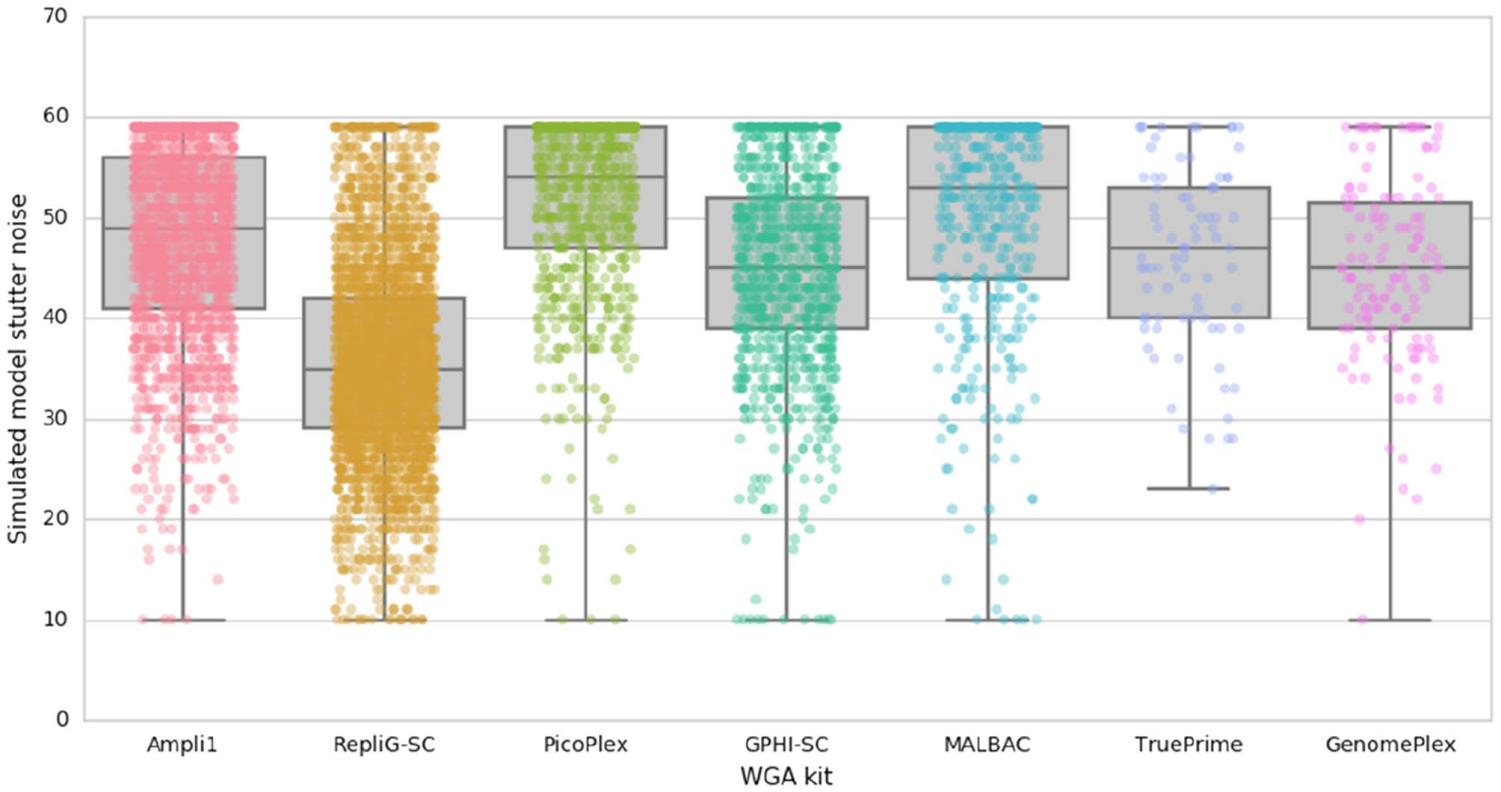
Error rate analysis of different scWGA kits. Simulated model stutter noise was fitted for AC type STR loci targets as part of the STR genotyping process^14^. RepliG-SC demonstrated the least stutter accumulation as expected from an MDA based method. PCR based protocols accumulate more relative stutter, equivalent to up to 20 additional PCR cycles. The targets were chosen from the X chromosome and covered with at least 30X reads, therefore a clear mono-allelic signal is expected. Indeed, high correlations between the model and measured stutter patterns are evident in Supplementary Fig. 3, regardless of the amount of simulated model stutter noise.

scWGA template is essentially a single genome copy (besides specific cell cycle periods^13^). Therefore, any in vitro mutation insertion, specifically at early stages of amplification, may lead to untraceable mutations that are eventually genotyped as real data. STR loci are prone for mutations caused during in vitro amplification^3^. In our previous work we have modelled the stutter patterns formed by STR in vitro amplification, and generated a novel STR genotyping tool^14^. This genotyping process compares the sequenced reads in the form of STR repeat count histograms against a library of modelled distributions, covering every possible repeat count within a specified range of amplification cycles. These libraries provided accurate matches at correlations exceeding 0.995 between the measured and the best fit model histogram. Each genotyping result provides not only the correct genotype (STR correct original repeat count) but also the modelled amplification cycle and a confidence score for the model matching. Here we used this tool to generate a measure of amplification noise for each STR in our panel (denoted: simulated model stutter noise). Since the downstream in vitro amplification is equal for every scWGA sample, the noise difference between different kits results from the scWGA reaction itself, and hence can be used as a comparative measure to determine the error-rate per kit. For this, we have analyzed only AC type STR loci, with more than 30 reads, from the X chromosome, to get a clear mono-allelic signal. We then plotted all simulated model stutter noise for all loci in all cells per scWGA kit (Fig. 3). RepliG-SC demonstrated the least simulated model stutter noise. This follows the expected consensus of MDA reduced noise over other methods (GPHI-SC, which is also MDA based is second, together with GenomePlex)^2^.

## Discussion

Large scale SC experiments are in increasing demand but choosing the correct WGA technology may not be derived by true comparisons of kits as such comparison is costly and laborious. Some comparative studies were previously performed, but they are either based on non-NGS analysis^9^, sequence non-eukaryotic cells^5^ or are limited by the number of cells per kit (< 9 cells, and in some cases only 2–3 cells)^6–8,10,12^. The high costs involved in SC genomics, which include the cost of scWGA reaction and costs of downstream analyses (e.g. whole exome sequencing (WES) or whole genome sequencing (WGS) of many cells) are the reason for the lack of large-scale comparison experiments.

In this study, we opted to conduct the largest scale (125 cells) scWGA kit comparison, containing, for the first time, all currently available commercial scWGA kits. We chose to analyze the scWGA products with a proven targeted sequencing benchmark analysis system^3^. To rule out batch effects we analyzed cells from the same clone and at the same day, and randomly distributed scWGA product in the analysis system PCR wells. Notably, improvements on existing kits were developed, namely upon MDA that is simpler than PCR based methods. These modifications require specific equipment^15,16^ or experimental design^13^ (cell stage or limited amplification). In this study, we therefore compared only commercially available kits and followed their manuals. Our experimental design advantages are: (a) cheap and therefore enables a large examined cohort of cells per kit, (b) comprises of a large amplicon panel (3401 amplicons) for improved statistics, and (c) relies on a genome template of a diploid normal cell line. This cell, when analyzed for X chromosome only, yields mono-allelic signal. We compared the following categories: genome coverage, reliability, reproducibility and error rate.

The reproducibility of a kit is sometimes of higher importance than of its genomic coverage. Phylogenetics algorithms compare the same data points (e.g. loci, SNPs) in every analyzed sample and later generate trees that reflect that comparison^17^. When analyzing SCs for their cell lineage relationship^18^, ADO plays an important role as it reduces the number of analyzed loci^17^. However, in such algorithms the comparable number of loci effect is even larger that the successful coverage per cell. For example: a data set of 70% genome coverage for two cells can range between fully reproducible loci number (70% of the data is comparable) to low reproducibility (until 40% comparable loci). Taking Ampli1’s internal bias against MseI containing amplicons (Supplementary Fig. 2) into account, it is the best kit in coverage and reproducibility. This biased amplification should be considered when planning an experiment. In most cases, one can order targeting probes/primers that consider Ampli1 biased amplification; however, this kit may not be suitable for several application types, and its protocol is much more laborious and not automation friendly as MDA based technologies.

In this experiment, PicoPlex was the most reliable kit, showing reproducible results for all cells, both in the coverage perspective and both in reproducibility perspective, with low variance for all analyzed cells (Fig. 2a,c). We chose to also present the data of the upper median of the most successful cells (Fig. 2b,d) as a simulation of a real experiment, where the best cells are chosen for analysis. In specific cases, such as rare cell populations, this selection is not an option. Moreover, the high cost per sample, ranging between 15 and 36$ per cell, makes the reliability improvement a key cost factor for a large-scale experiment. We believe that a fine calibration of every step of an experiment, from the cell picking, to the WGA procedure can achieve improved results for all kits. Results show that GenomePlex and TruePrime did not work in our hands. We suggest that further calibrations of their protocol may improve their results but was not in the scope of this study.

The current methodology to track and compare scWGA error rate is comparing the sequencing data generated from scWGA products to a reference genome, and therefore relies on a prior knowledge, which in the case of STR can be prone to errors or even not exist. We used our STR genotyping tool for “de novo” interpretation of error rate per locus without prior knowledge of assumption of its original STR length^14^. As expected, RepliG-SC excels as it is based on isothermal amplification that was previously described as having a low error rate than other WGA protocols^2^. This makes it favorable for variant analysis, specifically in SC experiments. GPHI-SC and TruePrime, which are also MDA based kits are amongst the three following kits, together with GenomePlex. Nevertheless, both TruePrime and GenomePlex have much less data points, due to their low success in this experiment. Although the starting template for PCR analysis was scWGA products, not normalized for their concentration, all of the kits manufacturers declare that the yield per SC is micrograms to tens of micrograms, a onefold difference. Since every PCR process yields sufficient amplification that presumably reaches a plateau, the difference between the amplification cycles per kit should be of maximum 3–4 cycles. The presented data on Fig. 3 simulated the number of noisy amplification cycles per kit. Even after addition of these 3–4 amplification cycles to RepliG-SC, it is still the best kit in the error rate aspect.

This study has several limitations (1) the experiment is limited to a targeted enrichment panel and is not a true random WGS experiment. The use of targeted enrichment as a subset of the genome is biased by its technology and by panel selection. In addition, in previous comparisons it was shown that with a low sequencing depth one could detect the coverage at a high significance^8^. However, even at a low depth of coverage, the cost per genome is not scalable to simulate a real SC experiment that usually comprises of tens to thousands of analyzed cells. The use of targeted sequencing therefore offers a cheap and reliable measurement that mimics a real experiment. (2) The panel is mostly comprised of STR containing amplicons that may bias the probability of amplification and affect the conclusions of the genome coverage and reproducibility results. A biased effect of this kind would result in a change in the composition of read counts per sample (STR containing amplicons and non-STR containing amplicons), compared with the original panel composition. To rule out this option we examined the above amplicon count composition of H1 bulk templates and compared them to the composition of the original panel. While the original panel composition is 95% and 5% (STR containing amplicons and non-STR containing amplicons, respectively), the compositions of amplicons count of H1 bulk template duplicate are 95.3% and 4.7% for duplicate 1, and 95.4% and 4.6% for duplicate 2, respectively, hence, amplicon count was not biased by amplicon composition. (3) One can choose to increase the panel size to improve statistics (e.g. exome panel). Increasing the probe/primers panel to larger genome panel will probably enable better statistical analysis. However, this will also dramatically affect the cost as in most cases, a change in targeted enrichment protocol will be required, and the cost per sequencing (of more bases) will also be increased. (4) Other cell properties cannot be detected by amplicon sequencing: uniformity analysis may be hampered as amplification is template sensitive, making its read coverage less informative for accurate original copy count inference *e.g.* for CNV profiling. Chimaeras, artefact joining of two separated genomic regions is also overlooked in amplicon sequencing: affiliated to MDA based analysis, chimaeras will not be detected as it will either not be amplified (if amplicon was not joined as a whole) or will be amplified without tracking of its occurrence.

It is clear from previous scWGA kit comparison experiments and from the data presented here that there is not a single winner in the race for the best scWGA kits, but several exceling kits, depending on the category of interest. Overall, this comparative assay demonstrates a cost-effective benchmark to compare different WGA kit properties of analyzed SCs and enables an educated selection of a WGA of choice, depending on the required application.

## Methods

### Generation of clonal human ES cells

H1 human ES cells (WA01) were obtained from the WiCell Research Institute (Madison, WI). In order to create SC clones, SCs were picked and deposited in separated single 96 wells using an automated cell picking device (CellCelector, ALS). Cells were cultured and treated as described in^3^.

### Cell picking and scWGA amplification

Followed by ~ 2 weeks of growth, a single clone was selected and detached to enable SC picking. For the cell picking procedure cells were treated as described in^3^ with the exception that prior to picking to scWGA reactions, cells were detached and dissociated using 550 U/ml StemPro Accutase Cell Dissociation Reagent (Gibco), incubated for 6 min, pulled down, re-suspended in iMEFs conditioned hESC medium (CM) and were spread over a 6 cm non-adherent petri dish.

At the day of the experiment bench and pipettes were decontaminated. Consumables (PCR plates, tubes, tips etc.) and pipettes were UV irradiated.

Individual cells were picked and deposited in separated single wells in scWGA kit dedicated 96-well PCR plates, pre-filled with each kit’s deposition buffer. All SCs were picked from the same clone and at the same day (besides GPHI-SC, which was picked a few days later). 1 µl of 100 pg/µl Hela genomic DNA (NEB) or water were added as controls to each reaction plate. Plates were directly processed with their appropriate scWGA protocol, avoiding any cell transfer during the process.

scWGA reactions were performed as recommended by the manufactures manuals (Table 1) with the following modifications, which were recommended by the manufactures to fit a > 2 µl deposition volume (cell picker requirement): (1) Ampli1—the deposition volume was modified to 5 µl PBS and Ampli1 Version 1 protocol was performed. (2) TruePrime—deposition volume was modified to 5 µl PBS, lysis was performed at 65 °C, and amplification mix contained 19.3 µl water, followed by a 3-h incubation. (3) GPHI-SC—the deposition volume was modified to 4 µl water. 1 µl lysis buffer (DTT 250 mM, KOH 1 M, Tween20 0.05%) was added to the reaction. Following lysis, 2.5 µl of Neutralization buffer were added. Amplification mix (composed of: 1.5 µl Enzyme Mix, 3 µl water and 16.5 µl Reaction Buffer) was added to each reaction, followed by 2 h of amplification at 30 °C and 65 °C inactivation for 10 min.

H1 cell bulk extraction (DNeasy Blood & Tissue Kit, Qiagen) served as a positive control to the targeted sequencing process.

### Processing DNA samples in the cell lineage analysis platform

We have utilized our lab developed cell lineage analysis platform as previously elaborated in^3^ to generate targeted enrichment NGS data from every cell sample. In short, scWGA samples, their controls and an H1 cell bulk sample (in duplicate) were randomized and placed in an AccessArray (AA) chip (Fluidigm) for targeted enrichment using 3401 primer pairs, divided to 48 multiplex reactions (See Supplementary Dataset 1 for description of primers and multiplex groups, and Supplementary Dataset 2 for description of all STR loci in the panel). Positive and negative controls (HeLa Genomic DNA 100 ng/µl and water, respectively) were added as additional controls in each of the 5 AA chips. AA PCR protocol was as in^3^. Barcoding PCR was modified from the original protocol to the NEBNext Ultra II Q5 Master Mix (M0544, NEB) protocol, using 0.5 µM primers and 0.5X SYBR green I (Lonza). Samples were purified and sequenced (Miseq, Illumina) after pooling in in an equal volume per sample (Echo550, Labcyte). Based on total reads per sample analysis, pooling using normalized volume was performed. Samples that did not yield enough reads were included in the pooling process such that: > 60% mapped reads were pooled at a fix volume of 6.5 µl, samples with < 60% and negative controls were pooled at the average volume of all normalized samples (667.5 nl). Sequencing was at ~ 100× per-amplicon depth (NextSeq, Illumina).

### Computational analysis

The STR-aware mapping of next generation sequencing was done using the pipeline described in Biezuner et al.^3^. STR mutation calling from repeat-number histograms was performed using the STR genotyping tool by Raz et al.^14^. The error rate analysis is based on AC type STR from X chromosome, with > 30 reads.

### Data access

Sequencing data generated in this study have been submitted to ArrayExpress (www.ebi.ac.uk/arrayexpress) under accession number E-MTAB-5968.

## Supplementary Information

Supplementary Information 1.

Supplementary Information 2.

Supplementary Information 3.

Supplementary Information 4.
