# Supplementary material for: Comparison of seven single cell whole genome amplification commercial kits using targeted sequencing
Source: Sci Rep. 2021 Aug 25;11:17171. doi: 10.1038/s41598-021-96045-9 (PMC8387353; doi:10.1038/s41598-021-96045-9)
Supplement: Supplementary file 1 — Supplementary Information 1. [file 41598_2021_96045_MOESM1_ESM.docx]

## Comparison of seven single cell Whole Genome Amplification commercial kits using targeted sequencing - Supplementary Information

Tamir Biezuner^1,*^, Ofir Raz^1^, Shiran Amir^1^, Lilach Milo^1^, Rivka Adar^1^, Yael Fried^2^, Elena Ainbinder^2^ & Ehud Shapiro^1^

^1^Department of Computer Science and Applied Mathematics, Weizmann Institute of Science, Rehovot 761001, Israel

^2^Stem Cell Core and Advanced Cell Technologies, Life Sciences Core Facilities, Weizmann Institute of Science, Rehovot 761001, Israel

*** Corresponding author**

Tamir Biezuner

Address: Department of Computer Science and Applied Mathematics

234 Herzl Street, Rehovot 7610001 Israel

Phone: +972-8-934-4494

E-mail: Tamir.Biezuner@weizmann.ac.il


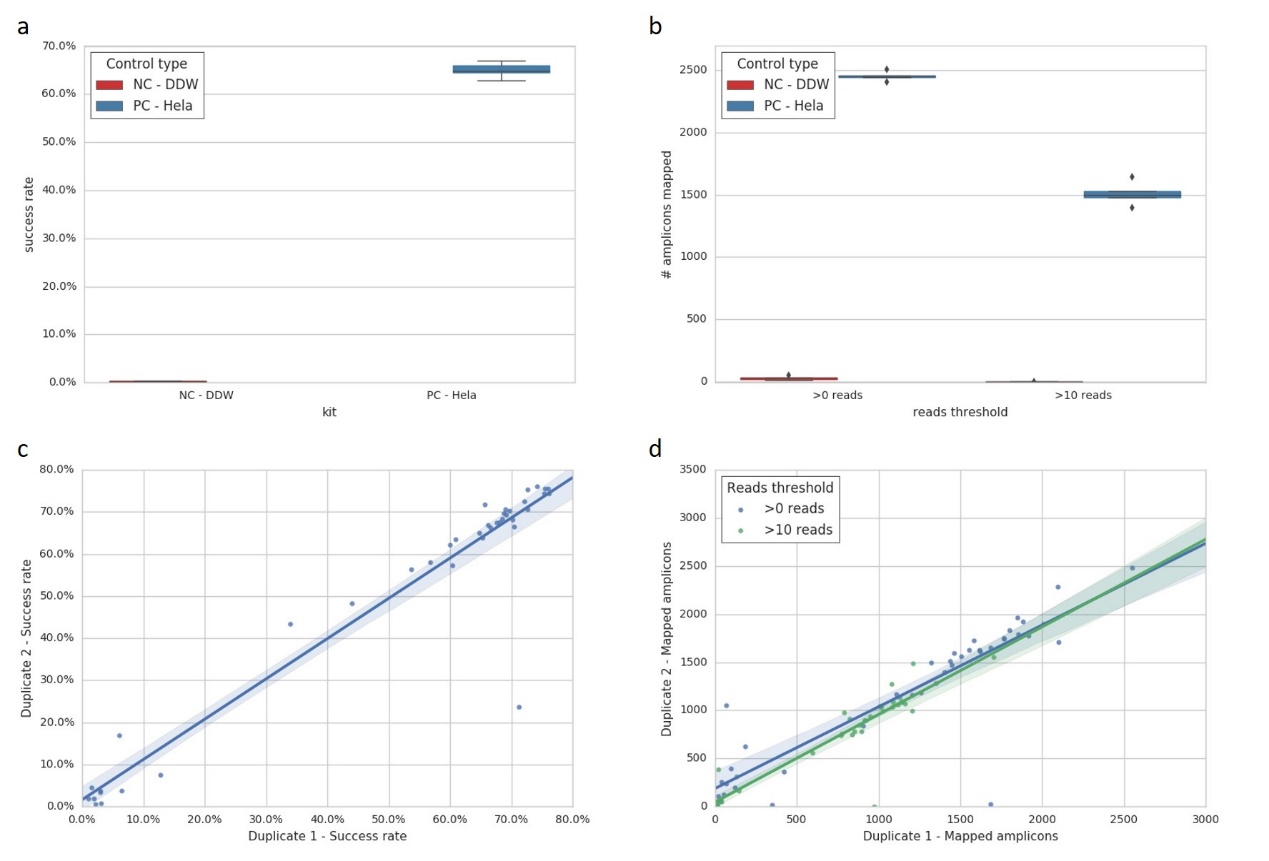


**Supplementary Figure 1. Experimental validation experiments.** (a, b) Reproducibility analysis by comparing negative (NC) and positive (PC) controls. Each was repeated 5 times, distributed between the 5 participating Access Array (AA) chips. (a) Success rate (mapped reads/ total reads per sample), and (b) count of amplicons with at least 0 mapped reads (left) and 10 reads (right). (c, d) Comparison between DNA duplicates. 39 Single cell duplicates and one cell bulk DNA duplicate were analyzed. Each DNA template was randomly distributed between the 5 AA chips. Duplicates were compared by the same analysis factors as in (a) and (b), respectively. The calculated coefficients of determination are for (c): R^2^ = 0.918 and for (d): 0 reads threshold R^2^ = 0.8 and 10 reads threshold R^2^ = 0.85.


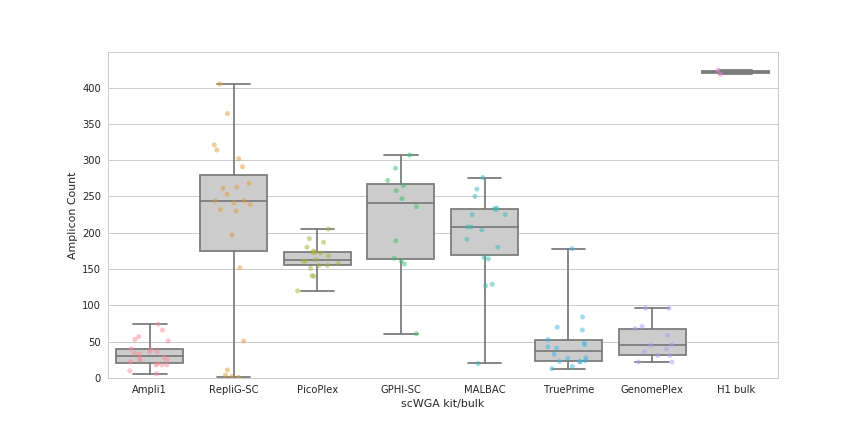


**Supplementary Figure 2.** **Amplicon coverage per single cell kit of only amplicons containing MseI restriction site (“TTAA”).** Mapped amplicons were counted per each single cell. Each dot represents a single cell, except for the right column, where each dot represents a cell bulk duplicate, originated from the same cell line (H1). Each column is the collection of all single cells per scWGA kit (except for the H1 bulk column). The theoretical maximum is 583 amplicons that follow the X chromosome, “TTAA” containing criteria.


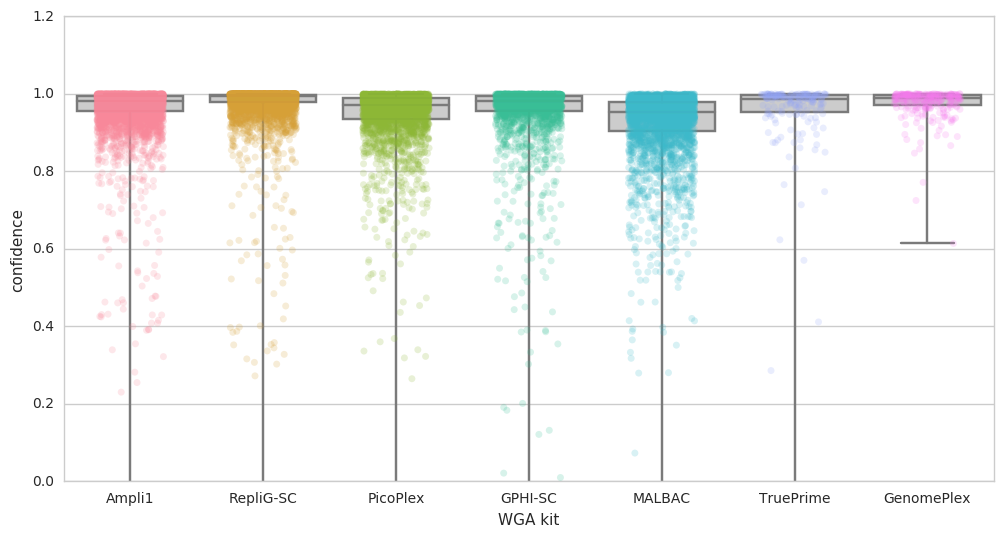


**Supplementary Figure 3. Confidence score of different scWGA kits.** AC type MS loci targets from the X chromosome (>30 reads) were analyzed using our MS genotyping tool^15^. This tool compares the stutter pattern observed in the NGS reads to simulated stutter patterns and estimates the amount of amplification (Figure 3). Genotyping confidence score was given to each analyzed locus and all analyzed scores per each kit were plotted, demonstrating that all kits presented the same quality of genotyping confidence.

**External files:**

**Supplementary Dataset 1. A detailed list of all 3401 amplicons.** Data includes amplicon ID, coordinates, corresponding PCR primers, the multiplex group each amplicon was part of (in the AA chip) and information regarding the existence of MS within the amplicons.

**Supplementary Dataset 2. A list of all MS targets and their coordinates.** Corresponding amplicons are details per target (some targets can be part of the same amplicon). Amplicons and multiplex groups are tagged by the same IDs as in Supplementary Dataset 1.

**Supplementary Dataset 3. A summary of mapped reads per sample per amplicon.** Each sample (rows) was mapped to the panel (columns). Numbers represent the count of mapped reads per amplicon per sample. Right side of table shows summary information regarding the mapping analysis (e.g. Total reads, success rate (mapped reads/total reads ratio), scWGA kit type).
